# Development of a Brain–Computer Interface Toggle Switch with Low False-Positive Rate Using Respiration-Modulated Photoplethysmography

**DOI:** 10.3390/s20020348

**Published:** 2020-01-08

**Authors:** Chang-Hee Han, Euijin Kim, Chang-Hwan Im

**Affiliations:** Department of Biomedical Engineering, Hanyang University, Seoul 04763, Korea; zeros8706@naver.com (C.-H.H.); u0038@daum.net (E.K.)

**Keywords:** electroencephalography, photoplethysmography, asynchronous brain-computer interface, respiration, steady-state visual evoked potential

## Abstract

Asynchronous brain–computer interfaces (BCIs) based on electroencephalography (EEG) generally suffer from poor performance in terms of classification accuracy and false-positive rate (FPR). Thus, BCI toggle switches based on electrooculogram (EOG) signals were developed to toggle on/off synchronous BCI systems. The conventional BCI toggle switches exhibit fast responses with high accuracy; however, they have a high FPR or cannot be applied to patients with oculomotor impairments. To circumvent these issues, we developed a novel BCI toggle switch that users can employ to toggle on or off synchronous BCIs by holding their breath for a few seconds. Two states—normal breath and breath holding—were classified using a linear discriminant analysis with features extracted from the respiration-modulated photoplethysmography (PPG) signals. A real-time BCI toggle switch was implemented with calibration data trained with only 1-min PPG data. We evaluated the performance of our PPG switch by combining it with a steady-state visual evoked potential-based BCI system that was designed to control four external devices, with regard to the true-positive rate and FPR. The parameters of the PPG switch were optimized through an offline experiment with five subjects, and the performance of the switch system was evaluated in an online experiment with seven subjects. All the participants successfully turned on the BCI by holding their breath for approximately 10 s (100% accuracy), and the switch system exhibited a very low FPR of 0.02 false operations per minute, which is the lowest FPR reported thus far. All participants could successfully control external devices in the synchronous BCI mode. Our results demonstrated that the proposed PPG-based BCI toggle switch can be used to implement practical BCIs.

## 1. Introduction

The brain–computer interface (BCI) is an emerging technology for providing patients that have neurodegenerative diseases with alternative communication channels by decoding neural signals voluntarily modulated to control external devices or generate messages [1]. BCIs have been developed using a variety of neuroimaging modalities, including electroencephalography (EEG) [2,3,4,5,6], magnetoencephalography [7,8], functional near-infrared spectroscopy [9], and functional magnetic resonance imaging [10,11,12]. Among these, EEG is most widely used because of its advantages over the other modalities, e.g., its cost-effectiveness, high temporal resolution, and portability [13,14]. Over the past decades, neuroscientists have developed various BCI paradigms based on specific EEG signal patterns, such as the steady-state visual evoked potential (SSVEP) [15,16,17,18,19], auditory steady-state response [20,21], event-related potential [5,22,23,24], slow cortical potential [25], and event-related synchronization/desynchronization [26,27]. These BCI paradigms can allow patients in a locked-in state to communicate.

EEG-based BCIs can be realized in either the synchronous or asynchronous mode [28]. The synchronous BCI is a traditional design in which information regarding the exact timing for the classification is provided. The users of synchronous BCIs can operate the BCI system only within a limited time period designated by operators. They cannot freely change the BCI operation mode, i.e., from the no control (NC) state to the intentional control (IC) state or vice versa. In contrast, the asynchronous BCI can detect the intention of the user without changing the operation mode and thus, is considered a better approach. Ideally, the users of asynchronous BCIs can use the BCI system whenever they wish, without time constraints.

Although there have been numerous attempts to realize practical asynchronous BCIs using various EEG patterns [28,29,30,31], most of them exhibited a high false-positive rate (FPR) in the asynchronous mode. One of the solutions for this problem is a two-step approach, in which the BCI system is first turned on using a “brain toggle switch” and then the intention of the user is identified in a synchronous BCI mode. EEG-based brain-switch systems have been introduced [32,33,34,35]; however, their FPR was too high to be used in practical scenarios. Please note that hereafter turning on a BCI system is defined as positive. Reducing the FPR is important because unwanted operation of BCI systems might sometimes cause dangerous situations, such as the malfunction of wheelchairs and robotic arms.

Physiological signals other than brain signals can also be used to implement a BCI toggle switch. Electrooculogram (EOG) signal patterns elicited by successive eye blinks have been widely used to toggle on and off BCI systems [36,37]. A recent study by Li et al. [36] reported an EOG-based BCI toggle switch system with an average accuracy of 99.5%, an average response time of 1.3 s, and an average FPR of 0.10 false positives per minute (FPs/min), which was tested with healthy subjects. Wang et al. [37] also developed a hybrid BCI utilizing EOG. Their hybrid BCI system combined motor imagery, P300, and eye blinking to implement forward, backward, and stop control of a wheelchair. Their EOG-switch based on eye blinking showed an average accuracy of 92%, an average response time of 2.0 s, and an average FPR of 0.30 FPs/min. The performances of these two EOG-based BCI toggle switches may look promising; however, the system might not be applicable to patients with severe amyotrophic lateral sclerosis (ALS), who are regarded as major target patients of EEG-based BCI technologies, because oculomotor function (including eye blinking) is generally impaired at the later stage of ALS [38]. Indeed, many ALS patients with impaired oculomotor function have difficulty in controlling their eyelid, but most of them can still use SSVEP-based BCIs [39]. Moreover, the FPR of 0.10/min is still high (six false operations per hour).

Herein, we propose a new BCI toggle switch that patients can use to toggle on or off the BCI by holding their breath for a few seconds. Inspired by the fact that respiration pattern is reflected in photoplethysmography (PPG) signals [40], features associated with breath holding (BH) were extracted from the respiration-modulated PPG signals. We used a PPG sensor instead of other respiration sensors (e.g., a respiration belt, temperature sensor, or CO2 sensor) because PPG sensors are significantly less expensive and easier to use. Moreover, there are many wearable PPG sensors available in the market; thus, the switch system can be readily incorporated with any type of BCI system. In this study, the implemented PPG switch was combined with an SSVEP-based BCI designed to control four external devices. When a user wanted to toggle on (or off) the BCI system, he/she simply needed to hold his/her breath for approximately 10 s. Then, two states—normal breath (NB) and BH—were classified using a linear discriminant analysis (LDA) in real time. The parameters of the PPG switch were optimized through an offline experiment with five subjects, and the performance of the switch system was evaluated in an online experiment with seven subjects.

## 2. Materials and Methods

In this section, we describe the experiments performed in the present study. Furthermore, we explain the overall procedure of the offline and online data analyses.

### 2.1. Participants

Five (all males, age: 25.80 ± 2.48 years) and seven (four males and three females, age: 23.86 ± 1.64 years) healthy subjects participated in our offline and online experiments, respectively. A comprehensive summary of the experimental procedure was provided to each subject before the experiments. The participants provided written informed consent and were reimbursed for their participation in the experiment. This study was reviewed and approved by the Institutional Review Board Committee of Hanyang University Hospital (HYUH 2015-11-031-001) and conformed to the tenets of the Declaration of Helsinki.

### 2.2. Offline Experiment

An offline experiment was designed to develop the respiration-modulated PPG switch. The following three issues needed to be addressed for developing the respiration-modulated PPG switch: (1) Can the PPG signals be used to differentiate the NB and BH states? (2) What is the best PPG feature for classifying NB and BH? (3) What is the optimal window size for classifying NB and BH using PPG signals?

To address these issues, PPG and respiration data were simultaneously recorded using a multi-channel biosignal acquisition system (ActiveTwo; BioSemi, Amsterdam, the Netherlands) at a sampling rate of 2048 Hz. The PPG signals were recorded using a finger-type PPG sensor (MLT1020FC, ADInstruments, Australia) attached to the left index finger of each participant, and the respiration data were measured using respiration belts fastened around the chests of the participants (SleepSense 1387-kit). Note that the respiration data were used as ground-truth data just to check whether participants performed the BH task well.

**Figure 1:**
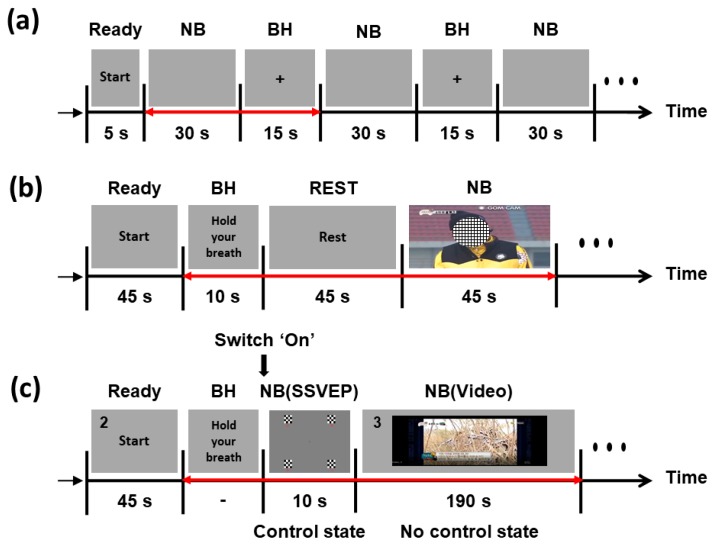
Schematics of the offline and online experiments: (**a**) offline experiment; (**b**) training session in the online experiment; (**c**) test session in the online experiment. The red double-sided arrows represent a single trial.

Figure 1a presents a schematic of the offline experiment. At the beginning of the experiment, a brief instruction was presented for 5 s to provide each participant with preparation time. Then, all the participants alternately conducted NB and BH tasks for 30 and 15 s, respectively. They were asked to rest during the NB periods and to hold their breath during the BH periods. During the entire experiment, their body movements were strictly limited to avoid potential movement artifacts. A set of NB and BH periods was repeated 20 times.

### 2.3. Offline Data Analysis

The PPG and respiration data were preprocessed using a series of signal-processing algorithms to remove unwanted artifacts. First, the PPG and respiration signals were bandpass-filtered at 0.2- and 0.4-Hz cutoff frequencies using a third-order Butterworth zero-phase filter. The frequency band of 0.2–0.4 Hz was selected, considering that the typical resting respiratory rate of a healthy adult is 12–18 breaths per minute (0.2–0.3 Hz) [41]. First-order differentiation was used to determine the slope values in the filtered PPG data. Finally, epochs corresponding to NB and BH periods were separately extracted (0–15 s for BH and 10–25 s for NB). The initial NB period (0–10 s) was not included in the epoch, because this period might have included deep breaths after the BH task.

To determine whether the PPG signals could be used to differentiate NB and BH, we compared the preprocessed respiration and PPG signals recorded while the participants performed NB and BH tasks.

To investigate the optimal feature for the classification of NB and BH states, a statistical analysis was performed using offline experimental data. Although the average respiration rate is approximately 0.3 Hz, individual variability may exist owing to several factors, such as age and health [42]. To select the optimal frequency range for differentiating NB and BH, the 0–1-Hz frequency interval was evenly divided into 0.0625-Hz-sized bins, and the average power spectral density (PSD) values were calculated for both the NB and BH states. The PSDs were obtained by an equation below. The PSD of a random time signals x(t) can be calculated using

(1) $$\underset{T\to \infty }{S_{x}\left(f\right)=\lim E}\left\{\frac{1}{2T}{\left|\int_{-T}^{T}x\left(t\right)e^{-j2\pi \mathrm{ft}}\mathrm{dt}\right|}^{2}\right\}$$

where E represents the expected value. Then, a paired t-test was performed to confirm the statistical significance between the two conditions.

Finally, pattern classification was performed to determine the optimal size of PPG data for classifying NB and BH. PSD values for each task were calculated in the optimal frequency range selected via the statistical analysis described in the previous paragraph and were used as a feature vector for the classification of NB and BH. Note that the number of features was just one. The LDA classifier was used to calculate the classification accuracy [43]. Then, the average offline classification accuracies were evaluated for different PPG data sizes using the leave-one-out cross-validation method, considering the relatively small number of task trials (20 trials for each task). Consequently, for each iteration, 38 and 2 trials were used for training and testing, respectively.

### 2.4. Online Experiment

The main objectives of our online experiments were to develop an online respiration-modulated PPG switch and to validate its performance with regard to the accuracy and FPR. PPG and EEG signals were measured using the same multi-channel biosignal acquisition device that was employed in the offline experiment. A Velcro-type PPG sensor (MLT1020PPG, ADInstruments, Dunedin, New Zealand) was attached to the left forearm of each participant using an adhesive disk. In the online experiments, we changed the location of the PPG sensor from the left index finger to the left forearm because it was reported that the forearm is better than a finger for measuring respiration-related PPG signal changes [44]. Three active electrodes (Oz, O1, and O2) were used to record EEG signals originating from the primary visual cortex, and the ground electrode was replaced with two electrodes—a common mode sense active electrode and a driven right leg passive electrode—both of which were attached in the central region (near CP1 and CP2, respectively). The offset voltage of all the EEG electrodes between the A/D box and the body was maintained between 25 and 50 mV, as recommended by the EEG device manufacturer. Both the PPG and EEG data were sampled at 2048 Hz.

The online experiment comprised an offline training session and an online test session. An LDA classifier for the PPG switch was trained using the PPG data recorded during the training session, and the performance of the PPG switch was evaluated in the online test session. Figure 1b shows a schematic of the training session. Before the training session, an instruction was provided for 5 s. The participants prepared for the training session during this period. After the instruction, each subject performed the BH task for 10 s, followed by 45 s of rest. We asked them to restore their NB during this rest period. Finally, each subject performed NB for 45 s while watching short video clips. This overall procedure was repeated three times. Figure 1c describes the test session. A start instruction for 45 s was provided to all subjects before starting the test session. At the end of the instruction, a beep sound was presented, and each subject performed the BH task until the PPG switch turned on the SSVEP-BCI system. If the SSVEP-BCI system was turned on, four-class SSVEP stimuli were presented for 10 s. We asked all subjects to gaze at a target stimulus during this period. Instruction for the target SSVEP stimulus was provided with a number during the start instruction period and the NB (video) period (see Figure 1c). The numbers were presented in the upper left corner of the monitor. After the SSVEP task, the NC state was provided for 190 s, and each subject watched a short video clip while performing NB. The time period of 190 s was introduced to monitor false operations of the system. These procedures were repeated five times.

In the four-class SSVEP-BCI paradigm, four visual stimuli were presented on a liquid-crystal display monitor. The distance between the monitor and the subjects was approximately 60 cm, and the refresh rate of the monitor was 60 Hz. Considering the refresh rate, four stimulation frequencies (6, 6.66, 7.5, and 10 Hz) were selected to elicit SSVEPs, and they were assigned to four different visual stimuli. A conventional black and white three-by-three checkerboard pattern was used as a visual stimulus and was reversed at different frequencies.

Four different environment devices (an electric fan, a heater, a lamp, and an emergency alarm) were used in the online experiment. When the subjects gazed at a target SSVEP stimuli, an environment device corresponding to the SSVEP-BCI result turned on or off. We controlled the environment devices by using Internet of Things-based smart plugs (SPW010P, Silo, South Korea).

### 2.5. Online Data Analysis

The raw PPG data were preprocessed using the same signal-processing methods that were employed in the offline analysis. Three 10-s epochs were extracted from the preprocessed PPG data for each of the NB and BH tasks. Because it was reported that the main frequency band associated with respiration does not depend on the PPG recording sites [44], we employed the frequency range of 0.2–0.4, which was determined in the offline data analysis. The PSD values in the frequency range of 0.2–0.4 Hz were calculated for each epoch, and this feature set was then used to train an LDA classifier.

While the respiration-based PPG switch was operating, the PSD feature was seamlessly calculated every second using the past 10 s of data. Simultaneously, the LDA classifier determined whether the participant was in the IC or NC state according to the classification outputs. The classification outputs were denoted by 1 or 2, representing NB and BH, respectively. Six consecutive classification outputs constituted a pattern, e.g., 111,221, 112,211, and 221,212, and the pattern was updated every second. The respiration-based PPG switch was turned on or off when the output pattern matched a predetermined template pattern (111,222 in this study). When a subject starts the BH task, the first classification is made using the 10-s PPG data recorded during the previous NB period, resulting in a classification output of ‘1’. After at least 5 s from the starting time of the BH task, the 10-s PPG data would have a chance to be classified as ‘2’. Therefore, at least 8 s are needed to make the target pattern of ‘111,222’.

When a participant turned on the BCI system by holding his/her breath, raw EEG data were recorded from three occipital electrodes (Oz, O1, and O2) for 10 s. We selected these electrodes because they have been widely employed in many previous SSVEP-based BCI studies [4,16,32]. The spectral powers at the four stimulation frequencies (6, 6.66, 7.5, and 10 Hz) for each electrode were calculated using a fast Fourier transform, with the window size set to be 10 s. In this procedure, EEG data from 0 to 1 s were rejected to minimize the potential motion artifact due to the deep breathing right after the breath-holding period. For the identification of the SSVEP stimulus that a participant was staring at, the spectral powers at four stimulation frequencies (6, 6.66, 7.5, and 10 Hz) and those at their second/third harmonics were summed over all three electrodes, and the frequency exhibiting the highest power value was selected.

## 3. Results

### 3.1. PPG Signal Modulated by Respiration

**Figure 2:**
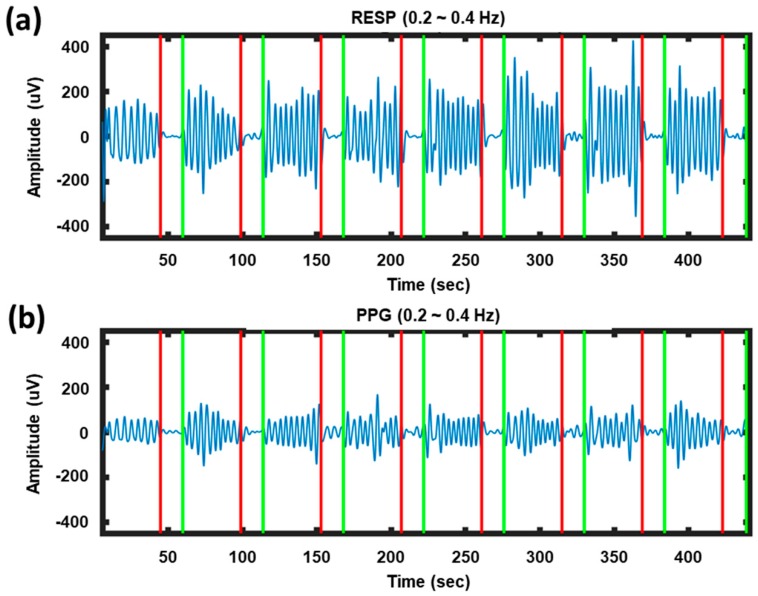
Examples of signals recorded while a participant alternately performed NB and BH tasks: (**a**) Respiration signals (denoted by RESP); (**b**) PPG signals. The red and green vertical lines indicate the onset times of BH and NB, respectively.

Figure 2 presents examples of respiration and PPG signals recorded while a participant alternately performed NB and BH tasks. The respiration and PPG signals exhibited similar patterns. When the participant performed the NB task, clear sinusoidal wave patterns were observed in the PPG signals as well as in the respiration signals. However, the sinusoidal nature of the waveform was diminished when the participant held his/her breath. This example indicates the possibility of using the respiration-modulated PPG signal as a toggle switch to turn on/off a BCI system.

### 3.2. PPG Feature for Classifying NB and BH

**Figure 3:**
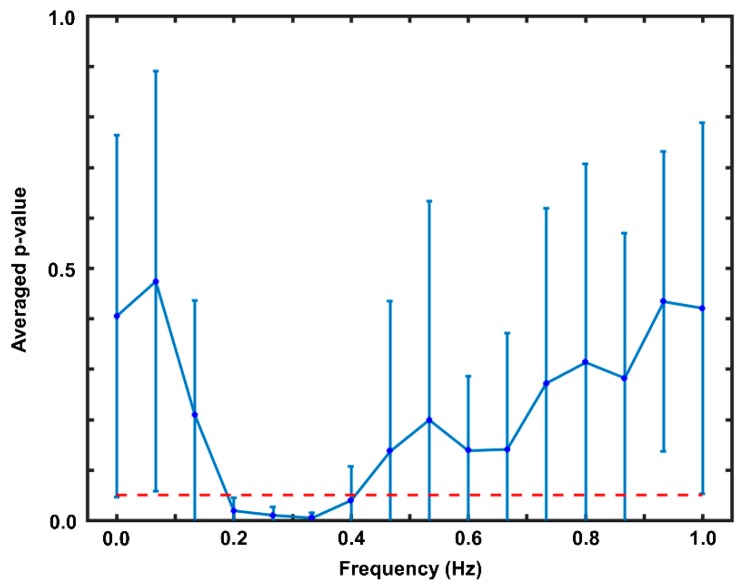
Results of the paired t-test in the offline data analysis. The red dotted horizontal line indicates a *p*-value of 0.05. The vertical axis indicates the *p*-value averaged across subjects in the offline experiment.

To identify PPG features for accurate classification of NB and BH, a frequency band having significantly different PSD values between BH and NB was selected via statistical analysis. Figure 3 presents the statistical analysis results. As shown, a significant difference between NB and BH was observed in the frequency band of 0.2–0.4 Hz (averaged *p*-value < 0.05). This frequency band was used to evaluate the PSD of the PPG signal, which was then used as a feature to classify NB and BH.

### 3.3. Optimal Time-Window Size

**Figure 4:**
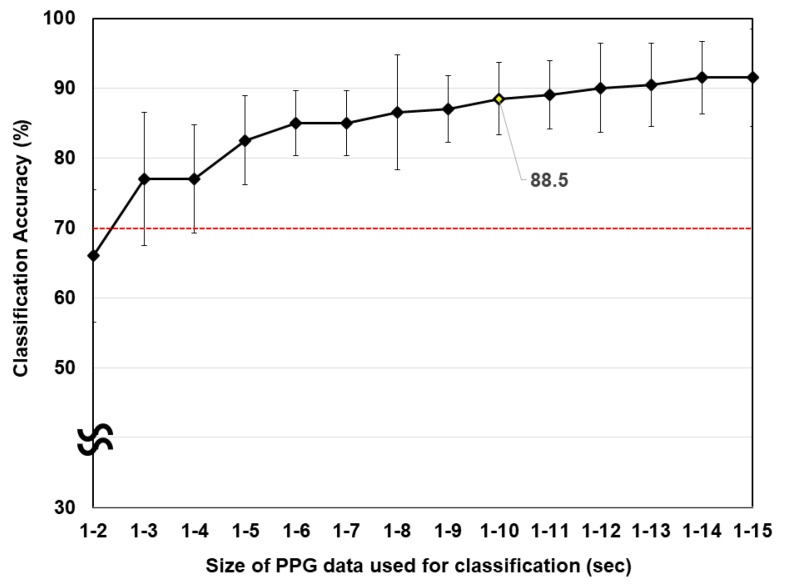
Average offline classification accuracy with respect to the size of the PPG data. The red horizontal line indicates the level of chance.

Figure 4 shows the classification accuracies with respect to size of the PPG data. The red dotted horizontal line represents the chance level. The appropriate level of chance for binary classification should be set as 70% when the number of trials is 20 and the confidence level is 99% [45]. PPG data from 0 to 1 s were excluded, considering the fluctuation of the PPG data immediately after BH. As shown in Figure 4, a larger window used for the classification yielded a higher classification accuracy. The offline classification accuracy was almost saturated when 10 s of PPG data were used for the classification of NB and BH (the classification accuracy reached 88.5%). Considering the tradeoff between the communication speed and classification accuracy, we used a 10-s window for the detection of BH in the subsequent online experiments.

### 3.4. Performance of PPG Switch

**Figure 5:**
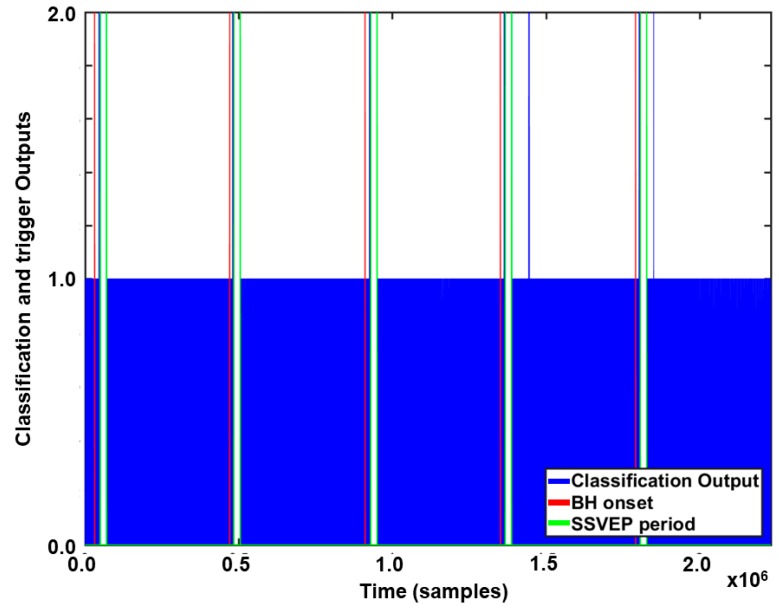
Example of online experimental results for a participant. The vertical axis corresponds to the classification outputs, which are indicated by the blue vertical lines. The red and green vertical lines indicate the onset times for BH and NB, respectively.

Figure 5 presents an example of the online experimental results. The vertical axis indicates the classification outputs, where 1 and 2 correspond to NB and BH, respectively. As shown in the figure, only two FPs were observed for five repeated trials.

Table 1 presents the overall online experimental results. All subjects succeeded in turning on the BCI system by holding their breath. The average elapsed time for turning on the BCI system was 10.57 ± 2.38 s. The true-positive rates (TPRs) were 100%. Furthermore, the FPRs were as low as 0.02 FPs/min. Five out of the seven participants did not exhibit any FPs. These results indicate that the proposed respiration-modulated PPG switch can be used as a reliable and robust BCI switch with an extremely low FPR and low inter-individual variability.

### 3.5. Online Control of External Devices

**Figure 6:**
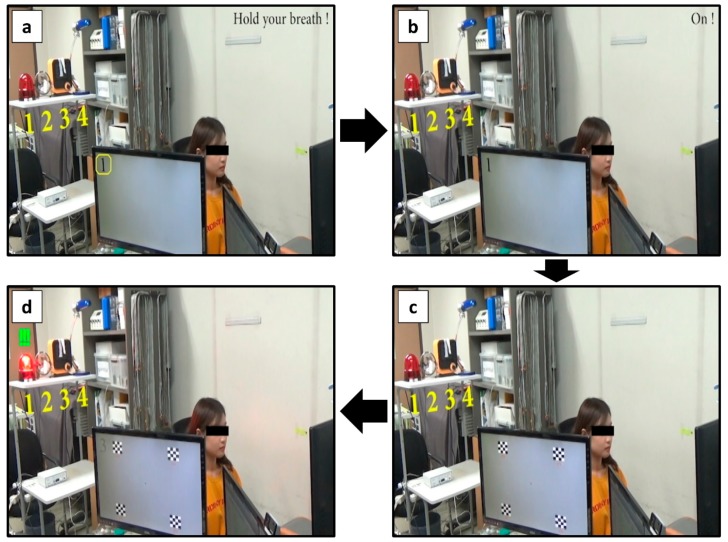
Series of snapshots taken during the online experiment with a healthy participant. (**a**) Participant held her breath until the PPG-based BCI toggle switch was turned on. (**b**) Once the PPG switch was turned on, auditory feedback (exclamation of the word “on”) was provided to her. (**c**) Immediately thereafter, four checkerboard stimuli with different reversing frequencies were presented for 10 s. During this period, she gazed at one of these visual stimuli. (**d**) After the classification, auditory feedback corresponding to the classification result was provided to her, and simultaneously, one of the environment devices was turned on.

Table 2 presents the online performance of the four-class SSVEP-based BCI, which was turned on by using the PPG-based switch. The target object was randomly presented to the participants, who were asked to operate one of the four external devices by staring at one of the pattern-reversal visual stimuli, which corresponded to an emergency alarm, a light, a heater, and a fan, respectively. After five repeated trials, the average accuracy was reported as 88.57%. Three out of the seven subjects achieved a classification accuracy of 100%. When the first 1 s of EEG data were included in the analysis epoch, the classification accuracy was dropped to 77.14%, suggesting that the first 1 s period might include some artifacts affecting the overall classification accuracy. Figure 6 presents a series of snapshots obtained during the online experiments when one of the participants successfully controlled one of the environmental devices. A video clip of the online experiment can be found in YouTube (see the following URL for more information: https://youtu.be/9KEcCh-evAA).

## 4. Discussion

We developed a respiration-modulated PPG switch with a low FPR and evaluated its performance by combining it with an SSVEP-based BCI. Users could turn on the BCI system using the PPG switch and could control four environment devices using the four-class SSVEP-based BCI. Our offline and online experimental results indicated that the proposed respiration-modulated PPG switch can be used for implementing asynchronous BCIs. To the best of our knowledge, this was the first study in which PPG signals have been used for implementing a BCI toggle switch.

Our respiration-modulated PPG switch exhibited a very low FPR (as low as 0.02 FPs/min), suggesting that users can operate our switch system for approximately 50 min without any false operations. The FPR of 0.02 FPs/min is the lowest FPR reported thus far for biosignal-based BCI toggle switches. EEG-based brain-switch systems [32,33,34,35,46] have the advantage that they do not require an additional signal-acquisition system other than the EEG; however, their FPRs are too high for application in practical scenarios. For example, a brain switch based on P300 exhibited a high FPR of 1.00 FPs/min [46]. SSVEP-based BCI toggle switches also exhibited high FPRs of 0.38 FPs/min [32] and 0.98 FPs/min [33], and motor-imagery-based BCI toggle switches exhibited a high FPR of 0.67 FPs/min [35] and 3.00 FPs/min [47]. In addition, hybrid BCI toggle switches, which simultaneously use two different BCI paradigms, showed 0.49 FPs/min [48] and 0.15 FPs/min [49]. EOG signals elicited by successive eye blinks can also be used for implementing a BCI toggle switch. A recently introduced EOG-based BCI toggle switch exhibited a high accuracy of 99.5% and a short response time of 1.3 s [36]. However, its FPR (0.10 FPs/min) was high compared with that of our respiration-modulated PPG switch. More importantly, this system might not be applicable to late-stage ALS patients, who generally have impaired oculomotor function. Our BCI toggle switch system not only has a very low FPR of 0.02 FPs/min but also is based on a straightforward respiration task. The users of our toggle switch are asked to simply hold their breath for a few seconds. In general, patients whose respiratory function is not impaired can easily hold their breath for a certain period of time; however, patients with ALS—who are among the major target users of EEG-based BCIs—gradually lose the ability to breathe voluntarily in the late stage of the disease. Artificial ventilation is generally used for patients with late-stage ALS [50]. Therefore, it should be confirmed whether patients with ALS can hold their breath for >10 s. To confirm this, we visited 10 ALS patients and measured their BH time using a stopwatch. The average R-ALSFRS [51] score of the ALS patients was 25.00 ± 14.42 out of 48. Two of the 10 patients had late-stage ALS, and their R-ALSFRS scores were just 1 and 4. The average BH time was 18.00 ± 4.11 s, and the two patients with severe ALS could also hold their breath for 10 and 20 s, respectively. The average BH time of approximately 18 s was long enough to use our respiration-modulated PPG switch, because the average time needed to turn on our BCI toggle switch was approximately 10 s. In the current system, some ALS patients might sometimes fail to turn on the BCI system; however, please note that they can still try the BH task once again after having a short rest. In addition, the average BH time is expected to be further reduced in future studies. The simplest way to reduce the time to turn on the BCI system is to shorten the length of the target pattern; however, this should increase the FPR. Therefore, new methods for BH detection, such as threshold-free methods [52] and dynamic-stopping approaches [53], should be considered in future studies.

Respiration signals can also be measured using a respiration belt as shown in Figure 2, where the average amplitude of respiration signals recorded from a respiration belt was larger than that from a PPG sensor. Although we might expect higher classification accuracy by employing a respiration belt instead of a PPG sensor, we employed the PPG sensor in this study because the PPG sensor is more convenient to use and easier to wear than the respiration belt. Indeed, it was difficult and often risky to put on the respiration belt to the ALS patients who were bed-ridden with mechanical ventilation. In addition, since most patients with ALS have severe respiratory muscle weakness, it is possible that the breathing belt can press the patient’s chest and disturb their normal respiration.

## 5. Conclusions

We developed a respiration-modulated PPG switch to implement an asynchronous BCI with a low FPR. Offline and online experimental results confirmed that the proposed PPG-based BCI toggle switch has a very low FPR of 0.02 FPs/min, indicating that a false operation occurs every 50 min. Additionally, all the participants in the experiments could successfully control four different environment devices by using the SSVEP-BCI incorporated with the PPG switch. It is expected that the proposed PPG-based BCI toggle switch can be used to implement asynchronous BCIs that can be employed in practical scenarios.

## Figures and Tables

**Table 1:** Online performance of the respiration-based PPG switch.

Sub	Time Elapsed for Turning Switch on (s)	TPR (%)	FPR (FPs/min)	Classification Accuracy (%)
1	8.03 ± 1.03	100	0.00	099.68 ± 0.47
2	10.17 ± 1.060	100	0.06	099.63 ± 0.82
3	9.91 ± 0.91	100	0.00	099.09 ± 0.79
4	11.69 ± 4.210	100	0.00	100.00 ± 0.00
5	10.41 ± 2.020	100	0.00	099.77 ± 0.52
6	12.29 ± 3.520	100	0.00	100.00 ± 0.00
7	11.49 ± 3.930	100	0.06	099.61 ± 0.87
AVG	10.57 ± 2.380	100	0.02	099.68 ± 0.50

**Table 2:** Online experimental results for the SSVEP-based BCI.

Subject	Order of Targets	Classification Results	Accuracy (%)
1	13,124	13,124	100
2	13,124	13,144	80
3	21,324	21,322	80
4	21,324	21,344	80
5	21,324	21,324	100
6	31,243	31,244	80
7	31,243	31,243	100
AVG	-	-	88.57
